# Supplementary material for: Synaptic vesicle release regulates pre-myelinating oligodendrocyte-axon interactions in a neuron subtype-specific manner
Source: Front Cell Neurosci. 2024 May 22;18:1386352. doi: 10.3389/fncel.2024.1386352 (PMC11150666; doi:10.3389/fncel.2024.1386352)
Supplement: Supplementary file 3 [file Data_Sheet_1.docx]

***Supplementary Figures***


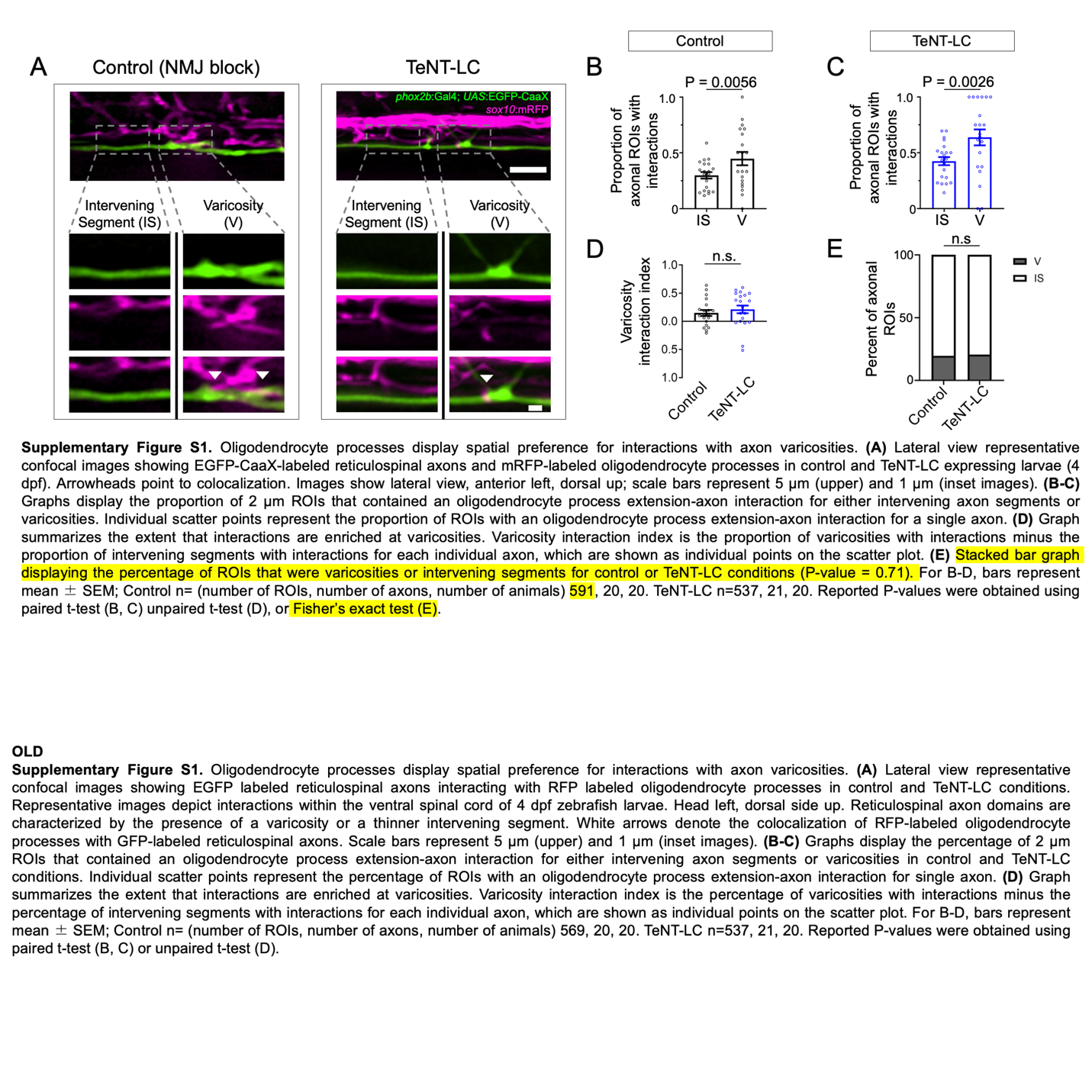


**Supplementary Figure S1. Oligodendrocyte processes display spatial preference for interactions with axon varicosities.** (A) Lateral view representative confocal images showing EGFP-CaaX-labeled reticulospinal axons and mRFP-labeled oligodendrocyte processes in control and TeNT-LC expressing larvae (4 dpf). Arrowheads point to colocalization. Images show lateral view, anterior left, dorsal up; scale bars represent 5 µm (upper) and 1 µm (inset images). (B-C) Graphs display the proportion of 2 μm ROIs that contained an oligodendrocyte process extension-axon interaction for either intervening axon segments or varicosities. Individual scatter points represent the proportion of ROIs with an oligodendrocyte process extension-axon interaction for a single axon. (D) Graph summarizes the extent that interactions are enriched at varicosities. Varicosity interaction index is the proportion of varicosities with interactions minus the proportion of intervening segments with interactions for each individual axon, which are shown as individual points on the scatter plot. (E) Stacked bar graph displaying the percentage of ROIs that were varicosities or intervening segments for control or TeNT-LC conditions. P = 0.7093. For B-D, bars represent mean ± SEM; sample size= (number of ROIs, number of axons, number of animals) 591, 20, 20 (control), 537, 21, 20 (TeNT-LC). Reported P-values were obtained using paired t-test (B, C) unpaired t-test (D), or Fisher’s exact test (E).


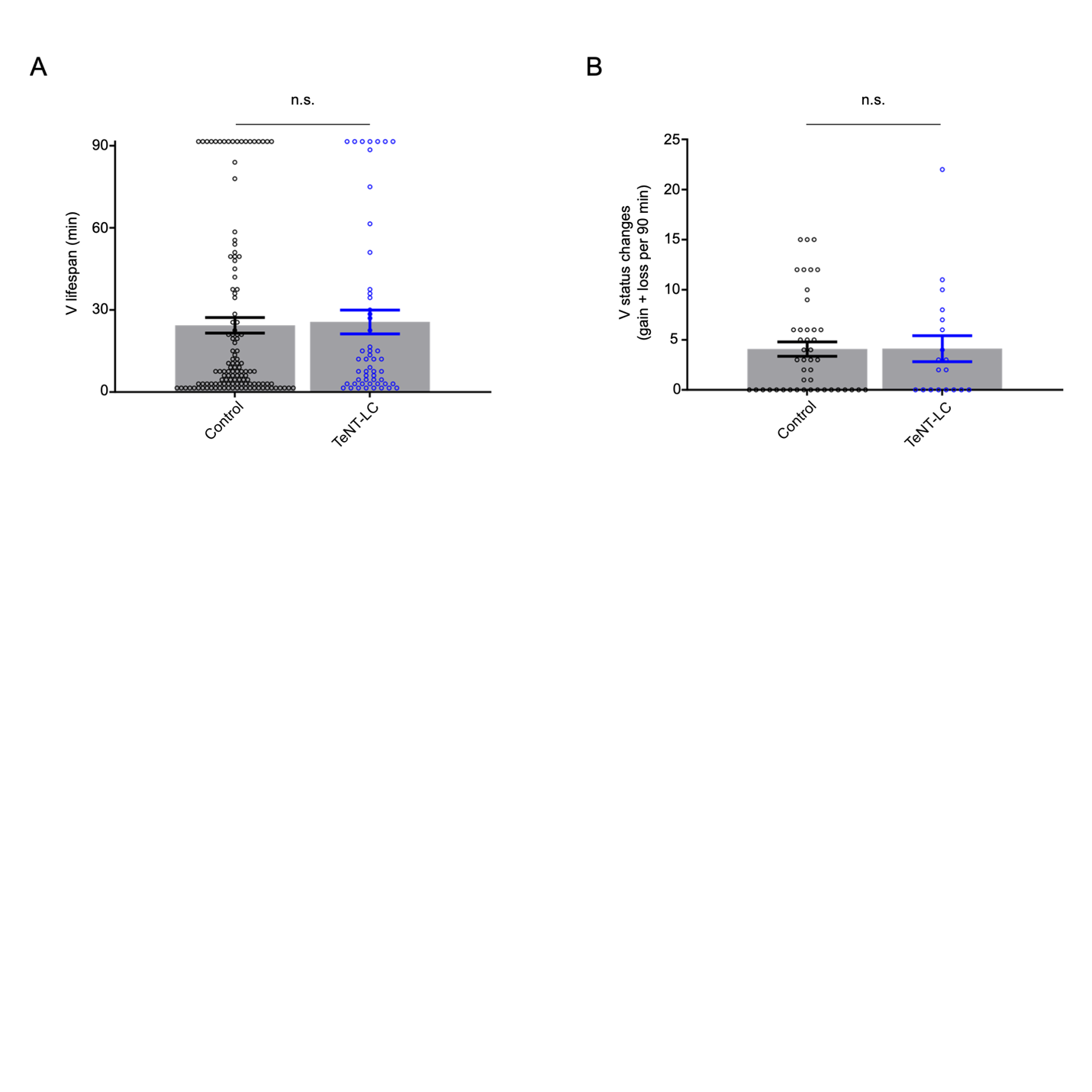


**Supplementary Figure S2. Axon varicosity lifespan dynamics are independent of synaptic vesicle release.** (A) Graph shows the length of time that individual axon varicosities persisted during the 90 min time-lapse. P = 0.4068. (B) Scatter plot points show the number of times that varicosities and intervening segments changed identity at individual axon segments during time-lapse imaging. P = 0.8532. For A-B, bars represent mean ± SEM. Sample size = (number of varicosities, number of animals) 45, 14 (control), 19, 9 (TeNT-LC). Reported P-values were obtained using the Mann-Whitney test.

**Supplementary Videos S1-S2. Time-lapse imaging of oligodendrocyte process extension-axon interactions.** Representative videos display a range of cell-to-cell behaviors observed in control conditions of Figure 1 datasets. Magenta shows *sox10*:mRFP labeled oligodendrocyte processes and green shows scatter labeled *phox2b* reticulospinal axons. **(Video S1)** Example of oligodendrocyte process extension interactions forming and retracting on a reticulospinal axon intervening segment. White arrows point at the site of RFP colocalization with GFP. **(Video S2)** Example of stable oligodendrocyte process extension interactions on a reticulospinal axon varicosity.
